# Prospective evaluation of testing with baked milk to predict safe ingestion of baked milk in unheated milk-allergic children

**DOI:** 10.1186/s13223-016-0162-9

**Published:** 2016-10-24

**Authors:** Allison Kwan, Maria Asper, Sasson Lavi, Elana Lavine, David Hummel, Julia E. Upton

**Affiliations:** 1 University of Toronto, Toronto, ON Canada; 2 Department of Pediatrics, Division of Immunology and Allergy, The Hospital for Sick Children, Toronto, ON Canada; 3 Department of Pediatrics, Humber River Hospital, Toronto, ON Canada; 4 Queen’s University, Kingston, ON Canada

**Keywords:** Cow’s milk allergy, Baked milk, Skin prick testing

## Abstract

**Background:**

Cow’s milk allergy is one of the most common food allergies affecting young children. A subset of milk-allergic individuals can eat baked milk without allergic symptoms which is beneficial in terms of prognostication and liberalization of the diet. A retrospective study suggested that skin prick testing (SPT) with a baked milk (muffin) slurry may provide a sensitive means of predicting the outcome of a medically supervised baked milk oral food challenge. We evaluated the predictive value of SPT with baked milk to identify unheated milk-allergic children who are able to safely eat baked milk.

**Methods:**

Children aged 2–16 years with a prior history of reaction to milk and a milk extract SPT of 8–14 mm were recruited. Investigator-blinded SPT to muffin slurry and powdered milk in triplicate and specific IgE (sIgE) to casein and milk were performed. Graded oral challenge to egg-free baked milk muffins (total 2.6 gm milk protein) was performed in the hospital. Reliability of tests was analyzed for intraclass correlation. Statistical significance for clinical characteristics of population and muffin testing versus baked milk reactivity was calculated with Fisher exact test for dichotomous and t-test for continuous variables. Wilcoxon rank sum test was used to compare immunological characteristics between individuals who tolerated or reacted to baked milk. Fitted predicted probability curves and ROC curves were generated.

**Results:**

Thirty-eight children were consented and 30 met study criteria. The muffin SPT and casein sIgE were significantly different in those who passed versus failed baked milk challenge. Negative (<3 mm) baked milk tests were found in 8/30 children (27 %) and were associated with non-reactivity to baked milk (p = 0.01) with a sensitivity of 1 (0.70–1.00). All children with negative SPT for baked milk passed the oral challenge. Specificity was 0.41 (0.19–0.67). The optimal decision point for the muffin SPT was 4 mm and the casein sIgE was 6 kU/L. The powdered milk test was not helpful.

**Conclusions:**

Skin prick testing with a baked milk (muffin) slurry may have a role in clinical practice to identify baked milk tolerance in milk-allergic patients.

## Background

Cow’s milk protein allergy is one of the most common food allergies encountered in clinical practice. It is predominantly seen in young children with estimates of up to 3 % of infants and young children affected [1]. Avoidance of all milk products has been the standard of care [2]. Due to the common availability of milk products, avoidance of not only foods made with milk, but also foods possibly contaminated with milk products, is difficult. In one study of young children, accidental exposures were responsible for 87.4 % of 834 accidental food allergy reactions and milk represented the highest annualized rate of reactions at 42.3 % of all reactions, both accidental and non-accidental [3]. Furthermore, almost one third of reactions requiring epinephrine administration were attributed to milk. Another study found that 15 % of young children with milk allergy had severe reactions resulting from accidental exposures over a 12 month period [4].

In recent years, evidence shows that 70–80 % of milk-allergic patients can eat baked milk products without reaction [5, 6], referred to as baked milk tolerance. This tolerance is due to conformational changes in milk proteins with whey more susceptible and casein more resistant to heat degradation [7]. In addition to extensive heating, there may also be an effect of the wheat matrix on milk allergenicity in baked products [8]. A study suggesting that there are at least two different phenotypes of IgE-mediated milk allergy in children demonstrates the clinical importance of tolerance to baked milk [5]. Only 9 % of children reactive to baked milk developed tolerance to unheated milk during the study period compared with 60 % of baked milk tolerant patients who developed complete resolution of cow’s milk allergy [9]. This study also suggested that inclusion of baked milk in the diet of children tolerant of extensively heated milk accelerates the rate at which milk allergy is outgrown. Furthermore, milk-allergic patients who tolerate baked milk are unlikely to present with a severe reaction when exposed to unheated milk [5]. It is therefore important to identify baked milk tolerant patients in the group of milk-allergic patients.

Physician-supervised baked milk oral challenges are recommended by leading allergists and are becoming common in clinical practice [10, 11]. Inclusion of baked milk to the diet reduces restrictions placed on patients with milk allergy.

Another approach under consideration for milk allergy is immunotherapy. Milk immunotherapy with unheated milk in a small study demonstrated that such intervention is efficacious in increasing the threshold for reaction [12]. However, a significant number of patients had allergic reactions during the protocol. Similarly, baked milk oral immunotherapy in baked milk reactive patients carried a high risk of reaction [13]. At present, such immunotherapy with milk remains experimental and is not recommended in clinical practice [14].

Oral food challenges are an important tool for assessing food allergy. These protocols are time consuming and patients should be selected appropriately. Reliable testing by skin prick and/or sIgE can significantly help clinicians in deciding on suitable candidates for oral challenge, either in hospital, the community, or at home. Widely used decision points to offer food oral challenges have been published for unheated milk [15, 16]. Sporik et al reported that a cow’s milk SPT of 8 mm was 100 % specific for unheated milk allergy in children over 2 years of age [15].

For baked milk, a retrospective study found that no patient with SPT <7 mm reacted to baked milk [6]. However, the study did not prove that patients were allergic to unheated milk. Larger tests resulted in decreasing probability of eating baked milk without reaction, but some patients with milk extract SPT as large as 20 mm were able to eat baked milk without reaction. Another study where patients were proven to be allergic to unheated milk found that no patient with a cow’s milk SPT <5 mm reacted to baked milk [5]. Almost 70 % of patients with a SPT 8 mm or greater could eat baked milk without reaction, but only 33 % of those with SPT 15 mm or greater tolerated the challenge. Given large variability, it is clear that improved testing is desirable, especially with children with intermediate results on milk extract SPT.

Cow’s milk sIgE has been evaluated for prediction of baked milk tolerance. Casein sIgE has also been studied given that it represents the major heat stable component of milk and may reflect allergy to baked milk. The higher the sIgE, the less likely a patient is to be able to eat baked milk without clinical reaction. The 95 % specificity level of cow’s milk and casein was determined in one cohort to be 24.5 and 20.2 kU/L, respectively [17]. However, a patient with undetectable sIgE to cow’s milk, including casein, can still react to baked milk [6]. Sensitivity of sIgE to casein is therefore less than optimal and medically observed oral food challenge is still required.

Studies to date have not identified a reliable commercial test to predict baked milk reactivity or tolerance. One retrospective study assessed whether skin testing with a muffin slurry could predict the outcome of an oral challenge [18]. Among 44 children, all 14 patients who did not demonstrate skin reactivity to muffin were baked milk tolerant. However, patients with positive muffin tests were not challenged and the average milk SPT in the cohort was small.

The objective of this study was to evaluate baked milk skin testing to see if it can predict those milk-allergic children who are able to safely eat baked milk. We chose a clinically relevant population of patients who had milk extract SPT testing large enough to likely have milk allergy but small enough to consider whether they could tolerate baked milk.

## Methods

Patients were recruited from both the community and the outpatient allergy consultation clinic at The Hospital for Sick Children for a prospective analysis of baked milk SPT to predict the outcome of oral food challenge to baked milk. Consent was obtained from guardians and assent from patients (where appropriate) in accordance with the institutional research ethics board (REB 1000042089). All SPT was performed with a stainless steel lancet (Medipoint) and in triplicate. SPT with commercial milk extract (Omega) and clinical history were used to determine study eligibility. Inclusion criteria were children >2 and <16 years of age, prior clinical reaction to unheated milk as defined by urticaria and/or angioedema or anaphylaxis, and positive SPT to milk extract ≥8 mm. Prior completion of sIgE was not a requirement. Patients were excluded if they had SPT to milk ≥15 mm, reacted to baked milk ingestion in the last 6 months, had risk factors for severe anaphylaxis (any of uncontrolled asthma or β-blocker or ACE-inhibitor use), eosinophilic disorders of the gastrointestinal tract or food protein induced enterocolitis syndrome, wheat allergy, significant skin disease, or prior determination of sIgE to milk or casein at or above a published 95 % specificity level [16].

Each muffin contained 1.3 gm milk protein (nonfat dry milk powder; Carnation, Smucker Foods of Canada), applesauce was substituted for egg, and muffins were baked at 350 °C for 30 min. SPT with a muffin slurry (1 gm of muffin in 10 mL of saline) and powdered milk (nonfat dry milk powder; Carnation, Smucker Foods of Canada, reconstituted as per manufacturer instructions) was performed and results were investigator-blinded. sIgE to milk and casein was obtained (ImmunoCAP, Phadia). Muffin oral challenges were performed openly under physician supervision. Challenges were discontinued at the first objective sign of reaction and patients treated accordingly. Muffins were administered incrementally over 75 min in six steps with 15 min in between. The dosing, as expressed as a fraction of one muffin, was 1/8, 1/8, 1/4, 1/4, 1/2 and finally 3/4, giving a total of two muffins (2.6 gm of milk protein). Patients were monitored throughout and for 3 h after final ingestion. Those who tolerated the challenge were instructed to include baked milk in the diet with written instructions based on published advice [10]. Subjects were contacted 1 month after oral challenge to assess compliance with incorporation of baked milk into the diet, and its tolerance.

Due to concerns about the homogenization of the muffin slurry, the reliability of the SPTs was analyzed for intraclass correlation. Statistical significance for clinical characteristics of the population and muffin test versus baked milk reactivity was calculated with Fisher exact test for dichotomous, and t-test for continuous, variables. Wilcoxon rank sum test was used to compare immunological characteristics between individuals who tolerated or reacted to baked milk. Fitted predicted probability curves were plotted using results from the logistic regression of the relationship between SPT or sIgE and food challenge outcome. Performance characteristics were obtained with ROC curves and the optimal cut-off was derived from the shortest distance to the point (0,1) on the ROC curve. Statistics were performed by a biostatistician with SAS 9.4.

## Results

**Fig. 1:**
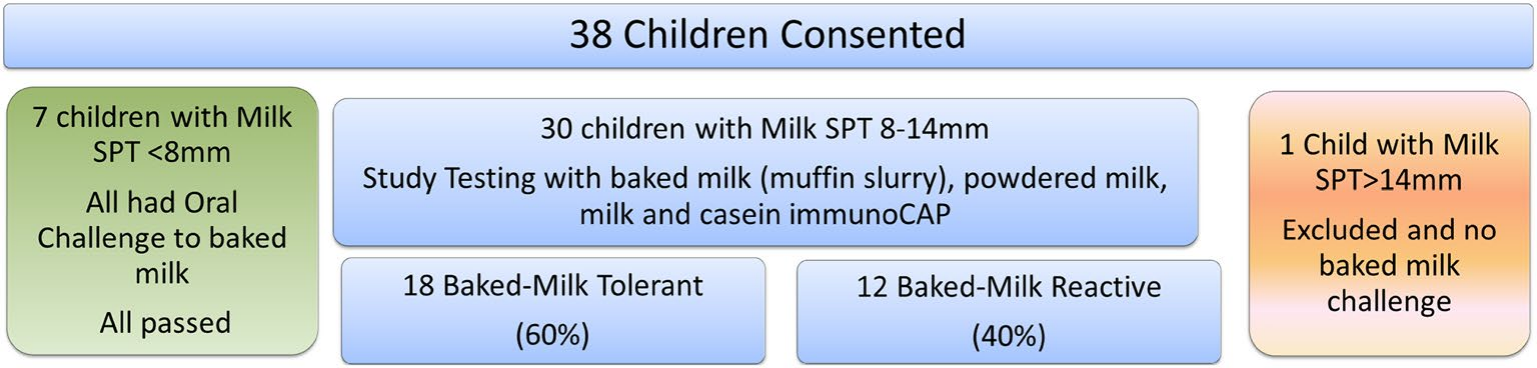
Study enrollment and outcomes of baked milk oral challenges. *SPT* skin prick test

Thirty eight children were evaluated at the Hospital for Sick Children and 30 children met inclusion and exclusion criteria. Seven children had milk SPT <8 mm; they were offered a baked milk challenge and they all passed. One child was excluded from the study on the basis of a milk SPT >14 mm. Of the patients that met inclusion criteria, the median age was 7.5 years old and 18 (60 %) were baked milk tolerant. The overall enrollment and baked milk challenge outcomes are shown (Fig. 1).

Overall, the challenges were well tolerated. Of the 12 participants who were baked milk reactive, 10/12 were treated with antihistamines and three required epinephrine, one for extensive urticaria which was very distressing to the child and the other two for wheezing.

At 24 h, 18/18 reported no symptoms and at 1 month follow up, 16/18 patients reported inclusion of baked milk in their diets without significant adverse reactions. One family did not answer the 1 month follow-up contact despite multiple attempts and another had not tried baked milk at home due to travel. One child had intermittent eczema which his mother thought might be related to baked milk but the family continued to include baked milk products in his diet.

Age, sex, asthma, eczema, time from last reaction to unheated milk, and prior history of anaphylaxis did not differ between those who were baked milk tolerant versus baked milk reactive (Table 1).

**Table 1:** Clinical characteristics of study population as related to baked milk reactivity

Patient characteristic (number of patients)	Passed baked milk challenge (n = 18)	Failed baked milk challenge (n = 12)	p value
Age, mean years	7.83	7.33	0.74
Male (24), Female (6)	14, 4	10, 2	0.99
Asthma (19)	10	9	0.44
Eczema (13)	8	5	0.99
Years since last reaction <1 (6), 1–2 (12), 3+ (12)	3, 5, 4	3, 7, 8	0.78
History of anaphylaxis (8)	5	3	0.99

The intraclass reliability of the muffin test was 0.822, similar to that of the commercial milk extract at 0.831. The size of SPT to muffin and sIgE to casein were statistically significant between the groups (Table 2). One sample intended for casein sIgE testing was not performed due to a sample processing error. The powdered milk SPT was not helpful. When analyzed as a dichotomous predictor, all children with negative (<3 mm) muffin SPT passed the baked milk challenge. Negative (<3 mm) muffin SPT were found in 8/30 children (27 %) and were associated with non-reactivity to baked milk (p = 0.01) with a sensitivity of 1 (0.70–1.00) and specificity of 0.41 (0.19–0.67).

**Table 2:** Immunological variables as related to baked milk reactivity

Immunological variable	Passed baked milk challenge median (min-max)	Failed baked milk challenge median (min-max)	p value
Muffin SPT, mm wheal	3.08 (0.0–13.8)	6.33 (3.83–8.33)	0.04*
Powdered milk SPT, mm wheal	9.5 (4.83–>20)	9.25 (5.5–17.2)	0.97
Milk sIgE, kU/L	6.91 (0.99–>100)	25.5 (1.82–>100)	0.10
Casein sIgE, kU/L	4.5 (0.35–>100)	19.7 (1.08–>100)	0.03*

* Significant

**Fig. 2:**
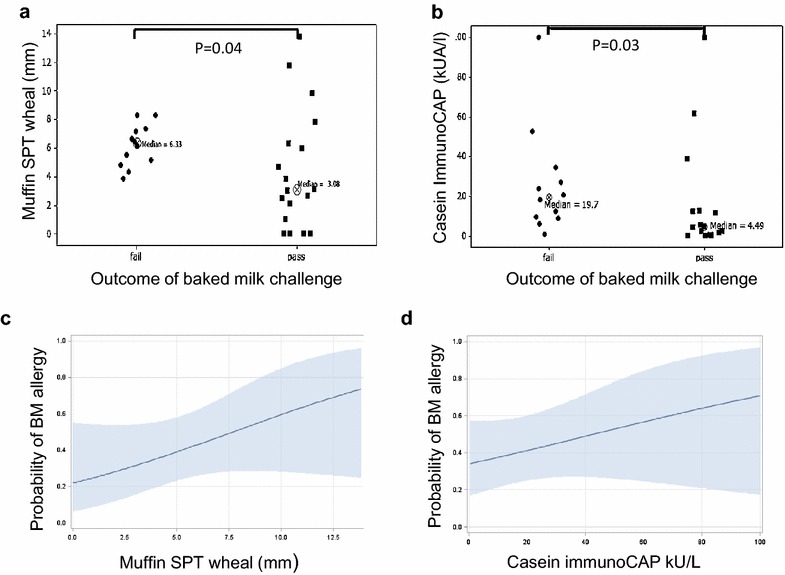
Outcomes and probability curves for Passing Baked Milk Challenges. **a** The size of the muffin test for participants that failed (reacted to) or passed the baked milk challenge. All individuals with a muffin test less than 3 mm passed the muffin challenge.** b** The casein sIgE test for participants that failed or passed the baked milk challenge.** c** The probability of being allergic to baked milk increased with larger muffin tests.** c** The probability of baked milk allergy increased with higher casein sIgE

Box plots and probability curves for passing a baked milk challenge are presented for the statistically significant tests of the muffin SPT and casein sIgE (Fig. 2). These curves show that both muffin SPT and casein sIgE can be used to predict the probability of baked milk reactivity.

Tables 3 and 4 present the performance characteristics of the muffin SPT and sIgE to casein. The optimal cut off point for the SPT to muffin was 4 mm. The optimal cut off point for casein sIgE was 6 kU/L.

**Table 3:** Performance characteristics of SPT to muffin slurry

Muffin test cutoff value (mm)	No at or exceeding cutoff	No failing challenge at or exceeding cutoff	No passing challenge at or exceeding cutoff	Sensitivity	Specificity	PPV	NPV
0.00	30	12	18	1.00000	0.00000	0.40000	1.00000
1.00	26	12	14	1.00000	0.22222	0.46154	1.00000
2.00	25	12	13	1.00000	0.27778	0.48000	1.00000
3.00	22	12	10	1.00000	0.44444	0.54545	1.00000
4.00^a^	18	11	7	0.91667	0.61111	0.61111	0.91667
5.00	15	9	6	0.75000	0.66667	0.60000	0.80000
6.00	13	7	6	0.58333	0.66667	0.53846	0.70588
7.00	8	4	4	0.33333	0.77778	0.50000	0.63636
8.00	5	2	3	0.16667	0.83333	0.40000	0.60000
9.00	3	0	3	0.00000	0.83333	0.00000	0.55556
10.00	2	0	2	0.00000	0.88889	0.00000	0.57143
11.00	2	0	2	0.00000	0.88889	0.00000	0.57143
12.00	1	0	1	0.00000	0.94444	0.00000	0.58621
13.00	1	0	1	0.00000	0.94444	0.00000	0.58621
14.00	0	0	0	0.00000	1.00000	0.00000	0.60000

^a^Optimal cutoff point for sensitivity and specificity

**Table 4:** Performance characteristics of casein sIgE

Casein sIgE cutoff value (kU/L)	No at or exceeding cutoff	No failing challenge at or exceeding cutoff	No passing challenge at or exceeding cutoff	Sensitivity	Specificity	PPV	NPV
0.00	29	12	17	1.00000	0.00000	0.41379	1.00000
1.00	24	12	12	1.00000	0.17647	0.5	1.00000
2.00	22	11	11	0.91667	0.29412	0.5	0.85714
3.00	20	11	9	0.91667	0.35294	0.55	0.88889
4.00	20	11	9	0.91667	0.47059	0.55	0.88889
5.00	19	11	8	0.91667	0.52941	0.57894	0.90000
6.00^a^	17	11	6	0.91667	0.64706	0.64705	0.91667
7.00	16	10	6	0.83333	0.64706	0.625	0.84615
8.00	16	10	6	0.83333	0.64706	0.625	0.84615
9.00	16	10	6	0.83333	0.64706	0.625	0.84615
10.00	14	8	6	0.66667	0.64706	0.57143	0.73333
20.00	9	6	3	0.50000	0.82353	0.66667	0.70000
30.00	6	3	3	0.25000	0.82353	0.50000	0.60870
40.00	4	2	2	0.16667	0.88235	0.50000	0.60000
50.00	4	2	2	0.16667	0.88235	0.50000	0.60000
60.00	3	1	2	0.08333	0.88235	0.33333	0.57692
70.00	2	1	1	0.08333	0.94118	0.50000	0.59259
80.00	2	1	1	0.08333	0.94118	0.50000	0.59259
90.00	2	1		0.08333	0.94118	0.50000	0.59259
>100.00	2	1		0.08333	0.94118	0.50000	0.59259

^a^Optimal cutoff point for sensitivity and specificity

## Discussion

All patients with negative skin testing to baked milk successfully tolerated an oral challenge to muffin. The study therefore confirmed the reliability of negative (<3 mm) baked milk skin testing in determining those children who are able to safely eat baked milk. However, allergic reactions to baked milk occurred with positive skin tests to the muffin slurry as small as 3.8 mm.

Our results are in keeping with those previously reported in a retrospective study where skin testing to extensively heated milk products was predictive of successful oral challenge to baked milk [18]. In that study some participants had small milk extract SPT and it was not known if they were truly allergic to unheated milk. In addition, patients with positive muffin tests were not challenged and the specificity was therefore unknown. Our study builds on the results of Faraj and Kim, reporting prospectively on a group likely to be allergic to unheated milk based on clinical history and skin testing. We also included patients with a positive SPT to baked milk.

Our study differs from those results of a study by Mehr et al [19] where baked milk skin tests <7 mm failed to show good negative predictive value. In that study the muffin was homogenized with normal saline [20] but the dilution of the muffin was not specified. The authors postulated that the high negative predictive quality of the baked cow’s milk muffin slurry as per Faraj and Kim [18] was confounded by inclusion of patients with small SPTs to milk. Addressing this gap in the literature, our patients were selected for a high likelihood of true cow’s milk allergy (milk SPT >8 mm and a previous clinical reaction to milk) and our muffin slurry was made in the same way as Faraj and Kim (1 gm muffin in 10 mL normal saline). We may have used a more dilute slurry than Mehr et al [19] which may account for our concordant finding with Faraj and Kim [18].

We confirm the finding that a positive test (≥3 mm) to the baked milk muffin slurry SPT has poor specificity. Very large tests (≥12 mm) had specificity approaching 95 % but the positive predictive value was poor in our population; the child with the largest test (13.8 mm) passed the baked milk challenge.

Consistent with other cohorts evaluating baked milk challenges, gender, history of asthma or eczema, and history of anaphylaxis were not able to clinically predict outcome of oral challenge [5, 6, 21]. Bartnikas et al [6], however, reported that younger children were more likely to be baked milk reactive. Such data is in contrast to the findings of Mehr et al [19] where history of asthma and anaphylaxis to milk were significantly different in patients reactive to versus tolerant of baked milk.

Use of powdered milk in place of muffin or other baked milk products has been proposed as a more readily available and standardized means of evaluating baked milk tolerance for both skin prick testing and oral challenge. In a recent study, Cherkaoui et al [21] suggested that 4 gm of milk protein reconstituted from powdered milk was a safe method of baked milk challenge and found no greater rates of positive challenge compared to studies using muffins. However, wheal size of powdered milk skin prick tests did not have discriminatory ability and were overall quite large. This SPT finding was replicated in our study where powdered milk SPT measurements were found to be upwards of 9 mm. Our study population differs in that Cherkaoui et al [21] did not set an upper limit for milk extract SPT values. We did use one of the same brands of powdered milk. It remains to be evaluated whether further diluting powdered milk would result in the ability to differentiate between baked milk reactivity and tolerance. In addition, there may be a role of the wheat matrix in the SPT material, and it is not known whether specific brands or batches of powdered milk yield different SPT results depending on their processing methods.

We did not analyze the predictive value of the milk SPT as we studied only children with a narrow range of milk extract SPT between 8 and 14 mm. Based on the results of Bartnikas et al [6] and Kim et al [9], we offered challenges to the seven children with SPT too small to meet study inclusion and they were all baked milk tolerant.

The milk sIgE test did not meet statistical significance to discriminate between those reactive to and tolerant of baked milk in our cohort. The casein sIgE was previously reported by Caubet et al [17] to have 95 % specificity (positive decision point for reactivity to baked milk) at a level of 20.2 kU/L and 95 % sensitivity (negative decision point) at 0.94 kU/L. All patients in their study who had undetectable casein sIgE tolerated baked milk challenges and the optimal cutoff point was 4.95 kU/L. Our results showed a similar optimal cutoff point of 6 kU//L to casein, and all five of our patients who had a casein sIgE <1 kU/L passed the muffin oral challenge. Our smaller sample never reached a 95 % specificity, but our casein value for 94 % specificity was much higher than Caubet et al [17], in part because a few children with very high casein sIgE passed the baked milk challenge (Fig. 2).

We confirmed that most families went on to incorporate baked milk products into their child’s diet illustrating the high compliance with this important dietary modification. No families had any significant reactions at home.

Out study’s strengths include prospective design, participants who were highly and likely allergic to unheated milk, testing that was blinded to investigators, SPT that was performed in triplicate, and statistical examination of the reliability of the baked milk skin testing as part of our methodology. The high prevalence of baked milk allergy in our cohort (40 %) speaks to the selection of an appropriate group. The baked milk challenge outcome was confirmed with a 24 h follow up phone call and ongoing ingestion of baked milk was confirmed for almost all participants at 1 month.

The major limitation of this study was its small sample size, Although the cohort size is similar to a previous report by Bartnikas et al [6], only 6 of their 35 patients were reactive to baked milk and thus represented a less atopic group. An additional limitation was that we did not confirm unheated milk allergy in this population. However, we selected patients to be very likely allergic to unheated milk by only including those with a clinical history of allergy to milk and milk extract SPT between 8–14 mm.

An additional consideration of this study is the age group in this study. The median age in our 30 participants was 7.5 years old. In the community practice sample described by Faraj and Kim [18] the median age for the 14 children included in the baked milk testing was only 3.5 years old. However, the median ages in other studies has been similar to ours at 8.1 years old [6], 6.6 years old [9], 9 years old [21]. The older age group seen in most baked milk study populations must be considered when generalizing results to community practice. We did not see a difference in the age of those who were baked milk reactive or tolerant, which is consistent with existing literature. In Mehr et al [19] the median age was 7.3 for reactive and 4.5 years old for tolerant to baked milk, although this was not statistically significant. In Caubet et al [17] in which there were more than 200 children with milk allergy, the median age was 7.3 (cohort 1) and 8.0 (cohort 2) years old to be reactive to baked milk and 6.5 (cohort 1) and 7.5 (cohort 2) years old to be tolerant and these differences within the cohorts were not statistically significant.

An approach for practitioners looking to find patients at low risk of baked milk reactivity is to consider offering such challenges to children with small milk SPT [6]. This may be reasonable in a community clinic. Our study supports that when a commercial milk extract SPT is large, sIgE casein testing can select for those children unlikely to react to baked milk. If blood work is not acceptable to families or difficult to obtain, our study suggests that skin testing to baked milk can select patients at low risk for reaction to a baked milk challenge. Further prospective trials are required to generate data to appropriately risk stratify those children with cow’s milk allergy who are being considered for baked milk challenges.

## Conclusions

Skin prick test with a cow’s milk muffin slurry is a reliable and useful test in helping to determine those milk-allergic patients who are likely baked milk tolerant in a population with a high prevalence of baked milk allergy. Positive testing to muffin slurry had low specificity and was insufficient to rule in baked milk reactivity. In children with milk SPT between 8 and 14 mm, a negative skin test to muffin had high sensitivity, and therefore high negative predictive value, for baked milk allergy. About 1/3 of children in our study had negative cow’s milk muffin slurry SPT and all of these children could safely eat baked milk which demonstrates its utility in this population. Additionally, SPT to cow’s milk muffin slurry is a very easy test to perform. Therefore, testing milk-allergic children with a cow’s milk muffin slurry may have a role in clinical practice to triage children at low risk for baked milk allergy. However, baked milk allergy should not be diagnosed on the sole basis of positive muffin tests. Finally, children who react to baked milk may have anaphylaxis requiring epinephrine so the setting of challenge should be chosen carefully.

## Abbreviations

SPT: skin prick test
sIgE: specific IgE
